# LDH-A—Modulation and the Variability of LDH Isoenzyme Profiles in Murine Gliomas: A Link with Metabolic and Growth Responses

**DOI:** 10.3390/cancers14092303

**Published:** 2022-05-06

**Authors:** Masahiro Shindo, Masatomo Maeda, Ko Myat, Mayuresh M. Mane, Ivan J. Cohen, Kiranmayi Vemuri, Avi S. Albeg, Inna Serganova, Ronald Blasberg

**Affiliations:** 1 Department of Neurology, Memorial Sloan Kettering Cancer Center, 1275 York Avenue, Box 52, New York, NY 10065, USA; shindoum@yukioka.or.jp (M.S.); masatomo.maeda@tokushukai.jp (M.M.); mko@wyckoffhospital.org (K.M.); manem@mskcc.org (M.M.M.); k.vemuri@rutgers.edu (K.V.); aalbeg09403@med.lecom.edu (A.S.A.); serganoi@mskcc.org (I.S.); 2 Molecular Pharmacology and Chemistry Program, Memorial Sloan Kettering Cancer Center, New York, NY 10065, USA; Ivan.Cohen@pennmedicine.upenn.edu; 3 Department of Neurosurgery, Nozaki Tokushukai Hospital, Osaka 5740074, Japan; 4 Human Oncology and Pathogenesis Program, Memorial Sloan Kettering Cancer Center, New York, NY 10065, USA; 5 Department of Radiology, Memorial Sloan Kettering Cancer Center, New York, NY 10065, USA; 6 Gerstner Sloan Kettering Graduate School of Biomedical Sciences, Memorial Sloan Kettering Cancer Center, New York, NY 10065, USA; 7 Center for Cellular Immunotherapies, Perelman School of Medicine, University of Pennsylvania, Philadelphia, PA 19104, USA; 8 Department of Genetics, Rutgers University, New Brunswick, NJ 08901, USA; 9 Department of Medicine, Division of Hematology and Medical Oncology, Weill Cornell Medicine, New York, NY 10021, USA

**Keywords:** glioblastoma, LDH-A shRNA knock-down, LDH isoenzymes, LDH-A and LDH-B immunohistochemistry, tumor growth, immune-competent and incompetent host animals

## Abstract

**Simple Summary:**

Three different murine glioma cell lines were modified to downregulate the expression of the murine *LDH-A* gene using shRNA knock-down (KD), and compared to shRNA scrambled control (NC) cell lines. Successful *LDH-A* KD was confirmed by differences in the expression of LDH-A and LDH-B mRNA, protein and enzymatic activity, as well as their LDH isoenzyme profiles. mRNA expression data indicated that: (i) GL261 LDH-A KD cells may have an improved ability to metabolize lactate into the TCA cycle; and (ii) that GL261 LDH-A KD cells can upregulate lipid metabolism/fatty acid oxidation pathways, whereas the other glioma cell lines (CT2A, and ALTS1C1) do not have this capacity. The data suggest that GL261 LDH-A KD cells can develop/activate alternative metabolic pathways for enhanced survival in a nutrient-limited environment. Furthermore, LDH-A KD prolonged the doubling time of GL261 cells in culture and prevented the formation of subcutaneous flank tumors in immune-competent C57BL/6 mice. No differences between NC and KD cell proliferation (in vitro) or tumor growth in C57BL/6 mice (doubling time) were observed for CT2A and ALTS1C1 cells and tumors. Our results suggest that GL261 glioma cells (but not CT2A and ALTS1C1 cells) are pre-programmed with a capacity for activating different metabolic pathways with higher TCA cycle activity, and that this capacity is enhanced by LDH-A depletion, leading to a difference in growth and survival in an immune-competent environment.

**Abstract:**

Three murine glioma cell lines (GL261, CT2A, and ALTS1C1) were modified to downregulate the expression of the murine *LDH-A* gene using shRNA, and compared to shRNA scrambled control (NC) cell lines. Differences in the expression of LDH-A and LDH-B mRNA, protein and enzymatic activity, as well as their LDH isoenzyme profiles, were observed in the six cell lines, and confirmed successful *LDH-A* KD. LDH-A KD (knock-down) resulted in metabolic changes in cells with a reduction in glycolysis (GlycoPER) and an increase in basal respiratory rate (mitoOCR). GL261 cells had a more limited ATP production capacity compared to CT2A and ALTS1C1 cells. An analysis of mRNA expression data indicated that: (i) GL261 LDH-A KD cells may have an improved ability to metabolize lactate into the TCA cycle; and (ii) that GL261 LDH-A KD cells can upregulate lipid metabolism/fatty acid oxidation pathways, whereas the other glioma cell lines do not have this capacity. These two observations suggest that GL261 LDH-A KD cells can develop/activate alternative metabolic pathways for enhanced survival in a nutrient-limited environment, and that specific nutrient limitations have a variable impact on tumor cell metabolism and proliferation. The phenotypic effects of LDH-A KD were compared to those in control (NC) cells and tumors. LDH-A KD prolonged the doubling time of GL261 cells in culture and prevented the formation of subcutaneous flank tumors in immune-competent C57BL/6 mice, whereas GL261 NC tumors had a prolonged growth delay in C57BL/6 mice. In nude mice, both LDH-A KD and NC GL261 tumors grew rapidly (more rapidly than GL261 NC tumors in C57BL/6 mice), demonstrating the impact of an intact immune system on GL261 tumor growth. No differences between NC and KD cell proliferation (in vitro) or tumor growth in C57BL/6 mice (doubling time) were observed for CT2A and ALTS1C1 cells and tumors, despite the small changes to their LDH isoenzyme profiles. These results suggest that GL261 glioma cells (but not CT2A and ALTS1C1 cells) are pre-programmed to have the capacity for activating different metabolic pathways with higher TCA cycle activity, and that this capacity is enhanced by LDH-A depletion. We observed that the combined impact of LDH-A depletion and the immune system had a significant impact on the growth of subcutaneous-located GL261 tumors.

## 1. Introduction

Lactate metabolism in tumors has been intensively studied recently [1]: (1) lactate is now considered a potentially major energy source for many tumors, (2) a major gluconeogenic precursor and (3) it exhibits signaling function properties. In this study, three murine gliomas (GL261, CT2A and ALTS1C1) were explored with regard to the impact of LDH-A downregulation on tumor biology, since there has been limited information on the role of the LDH-A/lactate axis in tumors of brain origin [1,2,3,4]. Lactate formation in gliomas is associated with poor survival and contributes to the suppression of local immunity [5]. The relationship between LDH-A expression levels and GBM malignancy, using human glioma cells and its impact on proliferation and apoptosis, has been explored [6,7]. It has long been known that many human cancers have higher LDH-A levels compared to normal tissues [8,9,10]. It has also been shown that LDH-A plays an important role in malignant development, local invasion and metastases [10,11,12]. The LDH enzyme (EC 1.1.1.27, LDH) is composed of two proteins, LDH-A (predominantly found in skeletal muscle and many solid tumors) and LDH-B (predominantly found in heart muscle and brain). The LDH enzyme is a tetramer and exists in several different electrophoretic forms known as isoenzymes. They catalyze the same biochemical reaction but differ in their kinetic characteristics, physicochemical properties (different net charge) and response to inhibition by pyruvate [13]. The LDH tetramer consists of either A or M subunits and the tetrameric enzyme exists in two basic homo-tetrameric forms: (i) LDH5 (A4 or M4) containing 4 LDH-A subunits, and (ii) LDH1 (B4 or H4) containing 4 LDH-B subunits. In addition to homotetramers, LDH also exists in three hybrid forms, resulting in five structural entities that vary in expression levels in different tissues [10]. The LDH-A and LDH-B isoforms occupy the mitochondrial compartment, plasma membrane and cytosol [14]. Traditionally, LDH-A participates in converting pyruvate to lactate, whereas LDH-B has a higher affinity for lactate, converting lactate to pyruvate and facilitating the use of lactate as a carbon energy source [15]. More recently, it has become clear that lactate is both created and consumed under aerobic conditions, and serves as a link between glycolytic and oxidative metabolism [16]. Analyses of LDH-A and LDH-B expression levels in tumors have shown that LDH-A is highly expressed in most neoplastic tissues [17,18,19,20]. However, the role of LDH-B and its regulation is less explored [21,22].

The role of LDH-A and LDH-B in tumor biology is complex. The relationship between tumor cell growth, the balance between LDH-A and LDH-B, the effects on tumor cell metabolism and the tumor microenvironment (TME) is variable across different types of gliomas [23,24,25]. In this report, we study and compare three murine glioma cell lines and s.c. tumors following LDH-A knock-down (KD). We found that LDH-A KD resulted in a reduction in glycolysis (GlycoPER) of all three cell lines, whereas a significant increase in the basal respiratory rate (mitoOCR) was only observed in GL261 cells; this suggests that GL261 glioma cells are more energetically limited (with the lowest ATP production) compared to CT2A and ALTS1C1 cells. Furthermore, a bioinformatics analysis of mRNA expression data showed that: (i) GL261 LDH-A KD cells may have a less glycolytic dependency and an improved ability to metabolize substrates through the TCA cycle; and (ii) that GL261 LDH-A KD cells can upregulate oxidative phosphorylation pathways, while the other glioma cell lines do not have this capacity. These two observations suggest that GL261 LDH-A KD cells can develop/activate alternative metabolic pathways for enhanced survival in a nutrient-limited environment, and that specific nutrient limitations have a variable impact on tumor cell metabolism and proliferation. In an accompanying manuscript [26], we compare the same cell lines and the intracranial growth of these tumors.

## 2. Materials and Methods

### 2.1. Cells and Culture Conditions

The GL261 murine glioblastoma cell line was obtained from an NCI depository [27,28]. The ALTS1C1 (ALT in the Figures) murine glioblastoma cell line derived from SV40 large T antigen-transfected astrocytes was kindly provided by Dr. Chiang (Department of Biomedical Engineering and Environmental Sciences, National Tsing Hua University, Taiwan) [29] and the CT2A high-grade murine astrocytoma cell line was kindly provided by Dr. Seyfried (Biology Department, Boston College, Boston, MA, USA) [30]. These cell lines were cultured in DME media supplemented with 25 mM glucose, 10% FCS, 4 mM glutamine and penicillin/streptomycin. LDH-A KD (knock-down) and NC (negative control) cells, derived from each cell line, were grown in the same media and 2.5 mg/L of puromycin.

### 2.2. Generation of LDH-A Knock-Down and Control Cell Lines

GL261, CT2A and ALTS1C1 cells were transfected with SureSilencing shRNA plasmids (QIAGEN, Frederick, MD, USA) to specifically knock-down the expression of the mouse *LDH-A* gene, as described previously [11]. Stably transduced clones (KD cell lines) were developed, along with a control (NC) cell line bearing a scrambled shRNA. Based on our previous experience [12,31], we decided to use the most effective shRNA (shRNA-2) from the set of 4 shRNAs to develop LDH-A KD in murine glioma cells. Our previous experience in other cell lines determined that shRNA-2 resulted in the best *LDH-A* knock-down function in murine cells. Although shRNA-3 *LDH-A* knock-down was less effective, the phenotypic changes in cells and tumors were comparable to that obtained with shRNA-2 [12,31]. The transfection of GL261 cancer cells with shRNA-2 resulted in a significant knock-down effect for LDH-A (approximately 30% of that in wild type cells), while bulk CT2A and ALTS1C1 cells transfected with shRNA-2 had a less profound LDH-A knock-down (40–60%) detected by mRNA proteins levels. To enrich the level of LDH-A knock-down, we used a sub-cloning strategy for CT2A and ALTS1C1 cell lines, while LDH-A GL261 knock-down cells were used as a bulk [11].

### 2.3. Western Blotting

All immunoblotting experiments were performed as described previously [11,32]. Cell lines underwent protein extraction using RIPA buffer (Thermo Fisher Scientific, Waltham, MA, USA) with protease and phosphatase inhibitors cocktail (1:100, Thermo Scientific Halt Protease & Phosphatase Inhibitor Single-Use Cocktail). Protein concentrations were determined by Pierce BCA protein assay (Thermo Fisher Scientific, Waltham, MA, USA). The proteins in equivalent amounts (10–40 µg/well) were separated by electrophoresis in a NuPAGE gradient 4–12% Bis-Tris Gel (Invitrogen, Carlsbad, CA, USA) and were immuno-blotted with anti-LDH-A antibody (#2012S, Cell Signaling Technology, Danvers, MA, USA) at a 1:1000 dilution and anti-ß-actin antibody (#A2103, MilliporeSigma, Rockville, MD, USA, USA) at a 1:5000 dilution of antibodies. Bound primary antibodies were visualized with either appropriate horseradish peroxidase–conjugated secondary antibodies (1:2000) (Cell Signaling Technology, Danvers, MA, USA) using an enhanced chemiluminescence reagent (Western Lightning-ECL) or with an Eu-labelled antibody using ScanLater Western Blot Assay kit and SpectraMax ID5 (Molecular Devices, San Jose, CA, USA). 

### 2.4. LDH Enzyme Activity

Total LDH enzyme activity was assessed using the Cytotoxicity Detection Kit PLUS (LDH) (MilliporeSigma, Rockville, MD, USA as described previously [11]).

### 2.5. Measurement of Changes in Cellular Bioenergetics in Real Time

Glycolytic activity and oxygen consumption of tumor cells were measured using a Seahorse XF96 Extracellular Flux Analyzer, as described previously [12]. Data were normalized to the number of cells in each well. Data from 3 independent experiments were analyzed using Seahorse Wave Desktop Software and compiled together using GraphPad Prism 7. 

### 2.6. mRNA Gene Expression Profile Analysis

LDH-A knock-down was verified by two approaches. First, a quantitative digital droplet PCR (ddPCR) was performed for LDH-A and LDH-B by the Genomics Core Laboratory at MSKCC. For RNA purification, cells were grown for 48 h (exponential growth phase). RNA was isolated using the RNeasy total RNA isolation kit (QIAGEN, catalog no. 74104), following the manufacturer’s protocol. Second, RNA extraction, library preparations and RNA sequencing reactions were conducted at GENEWIZ, LLC. (South Plainfield, NJ, USA). Total RNA was extracted from frozen cell pellet samples using Qiagen RNeasy Plus Universal mini kit, following the manufacturer’s instructions (Qiagen, Hilden, Germany). RNA Sample QC, DNase treatment, library preparations, sequencing reactions, and read mapping and alignment were conducted at GENEWIZ, LLC. (South Plainfield, NJ, USA) and are described in the Supplemental Information. After the extraction of gene hit counts, the gene hit counts table was used for downstream differential expression analysis. Using DESeq2, a comparison of gene expression between the groups of samples was performed. The Wald test was used to generate *p*-values and Log2 fold changes. Genes with adjusted *p*-values of <0.05 and absolute log2 fold changes of >1 were called as differentially expressed genes for each comparison. Significantly differentially expressed genes were used for Gene Set Enrichment Analysis (GSEA) using the fgsea package in the R statistical software (v4.0). Gene sets (pathways) used were downloaded from the Broad Molecular Signature Database and only pathways from Gene Ontology (GO), Reactome or KEGG were used.

### 2.7. Proliferation Assay In Vitro

The 2 × 10^5^ cells were seeded in 3 mL culture media in 6-well plates, followed by counting cells at 3 different time points: 48, 72 and 96 h after seeding cells using Countess Automated Cell Counter (Thermo Fisher Scientific, Waltham, MA, USA). Each sample was triplicated, and media were changed every other day. 

### 2.8. Animal Models

The animal protocol was approved by the Institutional Animal Care and Use Committee of Memorial Sloan Kettering Cancer Center (protocol number: 08-07-011; approval date for presented data: 19 September 2014). Two strains of mice were used in the animal experiments. First, 1 × 10^6^ cells in 100 µL PBS were injected into the right flank of 4–6-week-old immunocompromised Hsd: Athymic Nude Foxn/nu female (Envigo, Indianapolis, IN, USA) or athymic *nu*/*nu* female mice (Charles River Laboratories, MA, USA). Second, 1 × 10^6^ cells in 100 µL PBS mixed with 100 µL matrigel were injected subcutaneously into the right flank of immunocompetent C57BL/6 male mice (Charles River Laboratories, Wilmington, MA, USA). The volume (*V*) of subcutaneous tumors was calculated from caliper measurements, where V = (π/6) × x × y × z where x, y, and z are 3 orthogonal diameters. Doubling times were calculated by the equation of trend lines using GraphPad Prism. 

### 2.9. LDH Zymography

Zymography, a common method to detect isoenzymes, was used to detect tissue-specific differences in LDH isoenzymes. This approach can directly observe 5 isozyme bands in the active state [33]. Based on their different electrophoretic motility, all LDH isoenzymes can be identified as LDH1 (B4 or H4), LDH2 (B3A1 or H3M1), LDH3 (B2A2 or H2M2), LDH4 (B1A3 or H1M3) and LDH5 (A4 or M4). The buffer system at pH 8.6 was chosen for the best separation of the five LDH isoenzymes [33,34,35,36]. Because the B polypeptide has more acidic amino acid residues than the A polypeptide, LDH1/B has the highest migration rate and LDH5/A has the lowest migration rate. The electrophoretic mobilities of the LDH isoenzymes are: LDH 1/B > LDH 2 > LDH 3 > LDH 4 > LDH 5/A. 

### 2.10. Immunohistochemical Staining and Image Analyses

Dissected tumors were placed into 4% paraformaldehyde for further immunohistochemistry (IHC). The immunofluorescent (IF) staining was performed at the Molecular Cytology Core Facility of MSKCC using Discovery XT processor (Ventana Medical Systems). The 5 µm thick, paraffin-embedded sections were stained for H&E and LDH-A and LDH-B staining. The sections of tumors from nude or immunocompetent mice were stained by anti-LDH-A and anti-LDH-B. The small 5-day tumors from GL261 NC and KD were stained with immune markers: anti-CD68 antibody (Catalog No. TA1518, Boster), anti-CD4 (Catalog No. AF554, R & D Systems) and anti-CD3 antibody (Catalog No. A0452, Dako). Quantification of morphological characteristics was performed using trainable Weka Segmentation (Image J segmentation plugin) to assess the fraction of viable tumor cells, stroma, hemorrhage and necrosis in the H&E sections. The same approach was used to quantify LDH-A and LDH-B staining [31,37].

### 2.11. Statistical Analysis

Results are presented as mean ± standard error unless otherwise specified. Statistical significance was determined by a two-tailed Student t-test. A *p*-value of <0.05 was considered significant. All data presented for T cells assessment using IF staining were analyzed using GraphPad Prism (version 7.0; GraphPad Software) and are presented as mean +/− SD. Results were analyzed using the unpaired Student’s t-test, and statistical significance was defined as *p* < 0.05. 

## 3. Results

### 3.1. Effects of LDH-A Knock-Down on Murine Glioma Cells

**Figure 1:**
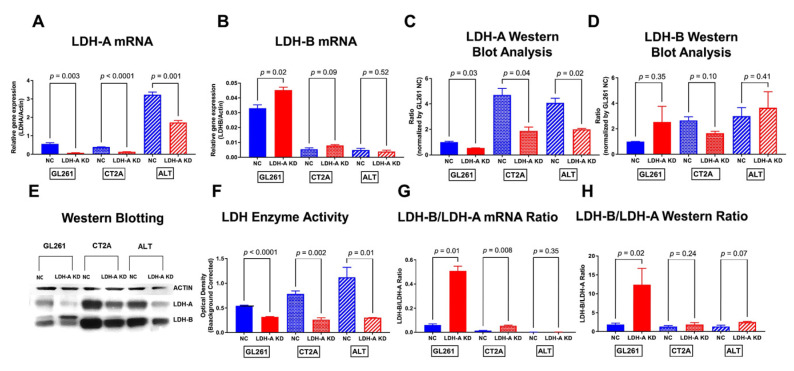
**Characterization of murine glioma cell lines (GL261, CT2A and ALTS1C1) following LDH-A shRNA knock-down**. LDH-A and LDH-B mRNA levels by ddPCR (**A**,**B**); protein expression on Western blot analyses (**C**–**E**); and LDH enzyme activity (**F**) in control NC and LDH-A KD cell lines (GL261, CT2A, ALTS1C1). LDH-B/LDH-A mRNA expression ratios (**G**) and LDH-B/LDH-A Western blot ratios (**H**) for control NC and LDH-A KD cell lines (GL261, CT2A, ALTS1C1). *n* = 3, ±SEM. The native Western blot for Panel E is shown in the Supplement (Figure S4).

**Figure 2:**
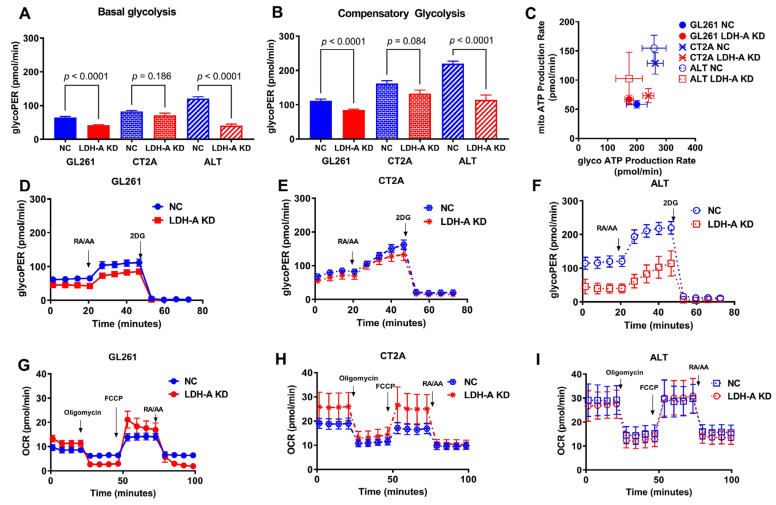
**Metabolic energy generation of NC and LDH-A KD murine glioma cell lines**. Glycolytic proton efflux rate (glycoPER): basal (**A**) and compensatory glycolysis (**B**) assessed by Seahorse XF analyzer (30,000 of NC and LDH-A KD GL261, CT2A, ALTC1S1 cells were seeded 4 h before the experiments). Results were normalized per 10,000 cells (n = 6, mean ± SEM); Rot/AA, rotenone + antimycin A; 2-DG, 2-Deoxy-D-glucose). Energy map of six tested cell lines charting mitochondrial ATP (mito ATP) versus glycolysis-generated ATP (glycol ATP) production; mean ± SD (**C**). Representative profiles of real-time glycoPER for GL261 (**D**), CT2A (**E**) and ALTS1C1 (**F**) cell lines, comparing NC and LDH-A KD. Representative profiles of a real-time OCR profile for GL261 (**G**), CT2A (**H**) and ALTS1C1 (**I**) cell lines, comparing NC and LDH-A KD. Values are mean, ±SEM; n = 5 (GL261); 4 (CT2A); 7 (ALTS1C1).

We chose three murine brain tumor models to understand the impact of LDH-A downregulation on tumor phenotype and growth potential: GL261 [27], CT2A [30] and ALTS1C1 [29]. LDH-A knock-down (KD) and control (NC) cells were obtained as described previously in the Material and Methods [12,31]. In vitro comparisons between the LDH-A knock-down (KD) and the scrambled control (NC) cell lines are shown in Figure 1, Figure 2 and Figure S1. Significant differences were observed between the three control (NC) cell lines with respect to LDH-A and LDH-B mRNA levels (Figure 1A,B and Figure S1A,B), LDH-A and LDH-B protein expression (Figure 1C–E and Figure S1C,D) and LDH enzyme activity (Figure 1F and Figure S1E). GL261 NC cells had the lowest expression of both LDH-A and LDH-B protein expression by Western blot assessment (Figure S1C,D) and have lower LDH enzyme activity (Figure S1E) compared with CT2A and ALTS1C1 NC cells. Despite low protein and enzyme levels, GL261 NC cells had the highest LDH-B mRNA level detected by ddPCR (Figure 1B and Figure S1B), and later confirmed by RNA sequencing data. This suggests an up-regulation of LDH-B in GL261 at the mRNA level but not at the protein level (possibly reflecting a lower rate of protein synthesis or more rapid protein degradation). Interestingly, CT2A NC cells had a comparatively low LDH-A mRNA expression (Figure 1A and Figure S1A) and a high LDH-A protein expression on Western blot (Figure S1C). These results suggest a variability in the synthesis vs. degradation of LDH-A and LDH-B between the three cell lines.

As expected, following LDH-A knock-down with shRNA, LDH-A mRNA levels, protein expression and enzyme activity were all significantly reduced compared to the control (NC) cell lines. The most notable difference was a significantly higher LDH-B/LDH-A ratio for mRNA, protein expression and LDH enzyme activity in GL261 KD cells (Figure 1F–H). 

### 3.2. Changes in Metabolic Properties of Glioma Cells Following LDH-A Knock-Down

To better understand the effect of LDH-A KD on the metabolism of the glioma cell lines and the effect of differences in the LDH-A/LDH-B ratio on the metabolic and phenotypic changes in glioma cells, we performed metabolic assays. The extracellular acidification rate (ECAR), the glycolytic proton efflux rate (glycoPER) and the oxygen consumption rate (OCR) were obtained, as described previously [12,31,38]. The injection of rotenone and antimycin blocks mitochondrial OXPHOS and provides an assessment of compensatory glycolysis (following the measure of basal glycolysis), and then by the addition of 2-DG to block glycolysis at the hexokinase step (Figure 2A,B,D–F). LDH-A knock-down resulted in a significant reduction in glycolysis (GlycoPER) in the GL261 and ALTS1C1 cell lines, but only a slight decrease in CT2A cells (Figure 2A,B,D–F). The Seahorse XF96 Analyzer also measures the real-time oxygen consumption rate (OCR, pMoles/minute), an indicator of mitochondrial respiration, in the same DME media (except for the absence of 5 mM HEPES) (Figure 2G–I). Using the cell mitochondrial stress test, we were able to measure basal respiration and maximal respiration, spare respiratory capacity and non-mitochondrial respiration using different modulators of cellular respiration [39]. The basal respiratory rate (OCR) and the maximal respiration capacity of LDH-A KD cells were considerably higher in GL261 KD and CT2A KD cells, but not ALTS1C1 KD cells, when compared to control NC cells (Figure 2G–I). Interestingly, both GlycoPER and mitoOCR were significantly lower in GL261 cells compared to CT2A and ALTS1C1 cells. To assess ATP production from mitochondrial respiration and glycolysis, total ATP production rates were obtained for all cell lines. This assay provided a measure of the energetic phenotype of the cells and allowed for a quantitative comparison of the impact that mitochondria and glycolysis have on basal energetic states (Figure 2C). These results indicate that GL261 glioma cells are more energetically limited compared to CT2A and ALTS1C1 cells, with the lowest ATP production by glycolysis and oxidative phosphorylation.

### 3.3. Changes in mRNA Expression Levels of Glioma Cells Following LDH-A Knock-Down

**Figure 3:**
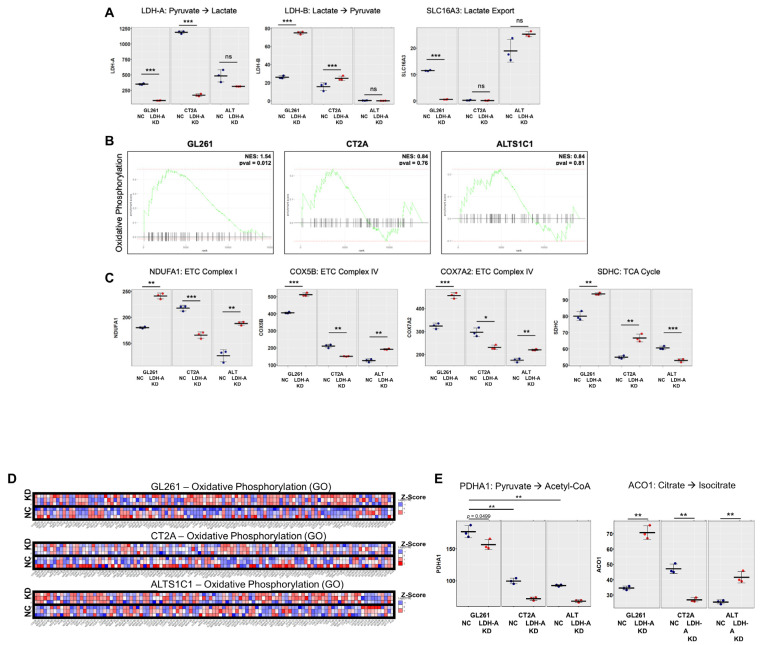
**Overexpression of genes involved in oxidative phosphorylation in GL261 LDH-A KD cells.** Transcripts per million (TPM) expression values were plotted in individual cell lines for genes directly involved in lactate metabolism and export (LDH-A, LDH-B, and SLC16A3) (**A**). Gene Set Enrichment Analysis (GSEA) for a single pathway (GO_OXIDATIVE_ PHOSPHORYLATION) is shown for each GBM cell line. The analysis shows the enrichment of this pathway in LDH-A-depleted vs. control cells (**B**). TPM values for enzymes involved in oxidative phosphorylation (**C**). The Z-transformed scores of individual genes within the oxidative phosphorylation (GO) pathway were plotted across each cell line (GL261, CT2A and ALTS1C1). The experiment was performed in triplicate, with rows representing each sample and columns representing individual genes (**D**). Expression (TPM) of pyruvate dehydrogenase alpha 1 (PDHA1) and aconitase 1 (ACO1) was plotted (**E**). PDHA1 is a nuclear-encoded mitochondrial matrix multienzyme complex that provides the primary link between glycolysis and the tricarboxylic acid (TCA) cycle by catalyzing the irreversible conversion of pyruvate into acetyl-CoA; ACO1 is a bifunctional, cytosolic protein that functions as an essential enzyme in the TCA cycle. Significant differences are indicated by: * *p* < 0.05, ** *p* < 0.01, and *** *p* < 0.001. Table S2 of the Supplement provides the semi-raw data from the RNASeq analysis that was used to generate the figures.

We performed RNA sequencing (RNASeq) to analyze the cellular transcriptome in all six cell lines (GL261, CT2A and ALTS1C1; both scrambled NC and LDH-A shRNA KD) to investigate changes in their transcriptomes. We confirmed LDH-A depletion in our LDH-A KD cell samples, and also observed a significantly increased expression of LDH-B in the GL261 KD cells compared to all other cells (Figure 3A). In addition to decreased LDH-A and increased LDH-B, GL261 KD cells also showed a significant downregulation of the lactate exporter MCT4 (*Scl16A3*). Given the increased OCR rates observed in GL261 and CT2A LDH-A KD cells (Figure 2), we performed Gene Set Enrichment Analysis (GSEA) for an oxidative phosphorylation pathway in each GBM cell line (GO_OXIDATIVE_PHOSPHORYLATION) (Figure 3B). This analysis showed that at the mRNA level, GL261 LDH-A KD cells had a significant enrichment of this pathway compared to NC. In addition, individual genes were chosen as an example; the expression of enzymes that make up the electron transport chain (ETC) and the TCA cycle are shown (Figure 3C,D). A similar GSEA for the glycolytic pathway showed little or no pathway enrichment of glycolytic genes comparing LDH-A KD to control NC cells (data not shown), although there was some enrichment of individual genes (e.g., PGK1 in GL261, ALDOA in CT2A and ENO1 in ALTS1C1 LDH-KD cells).

This suggested that GL261 LDH-A-depleted cells, through their unique upregulation of LDH-B, may be able to metabolize nutrients other than glucose. For example, lactate can be converted into pyruvate by LDH-B overexpression and support cell proliferation in nutrient-limited conditions, such as those found within subcutaneous-located solid tumor microenvironments. In support of this, we observed that GL261 cells (NC and KD) had the highest levels of *PDHA1* (pyruvate dehydrogenase E1 subunit alpha 1), which catalyzes the conversion of pyruvate into acetyl-CoA, which may further be used in the TCA cycle or for fatty acid synthesis (Figure 3E). 

### 3.4. Effects of LDH-A Knock-Down In Vitro and on Tumor Growth Profiles in Immune-Competent and Incompetent Animals

**Figure 4:**
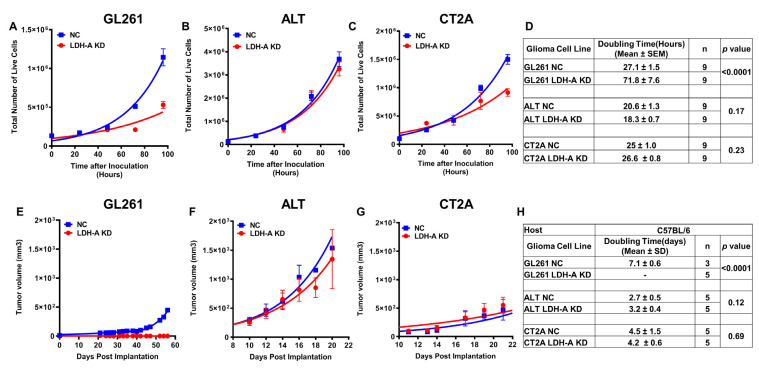
**The effect of LDH-A knock-down on cells in vitro and s.c. tumor growth, in vivo in immune-competent mice**. Growth profiles and doubling times of GL261, ALTS1C1 and CT2A cells in vitro (Panels **A**–**D**) (mean ± SEM) and tumors in C57BL/6 mice (Panels **E**–**H**) with and without LDH-A shRNA knock-down (mean, ± SD). Note that the doubling times for GL261 NC tumors (Panel E) were estimated after the initial delay in tumor growth (0~40 days).

The effect of LDH-A knock-down on cell proliferation (in vitro growth profile) was compared to that of control NC cells (Figure 4A–C). The cell proliferation rate (doubling time) in optimal nutrient media was calculated from an exponential fit of the plots (Figure 4D). The doubling time was prolonged (slower growth) only for GL261 LDH-A KD cells; there was little or no effect of LDH-A KD on CT2A and ALTS1C1 cells. The doubling times of wild type and NC cells were similar (data are not shown). The differences in the proliferation of cell lines suggest that there are corresponding differences in their metabolic properties, since metabolism and proliferation share common regulatory pathways in cancer cells [40,41,42]. 

**Figure 5:**
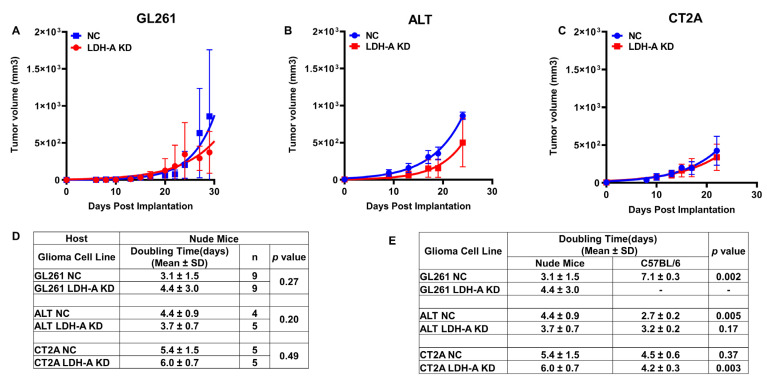
**The effect of LDH-A knock-down on s.c. tumor growth in nude mice**. Growth profiles of GL261, ALTS1C1 and CT2A tumors (Panels **A**–**C**), and estimated tumor doubling times (Panel **D**). Mean, ± SD. Comparison between tumor doubling times in nude mice and C57BL/6 mice (Panel **E**).

To further study the differences in tumor growth and phenotype, subcutaneous (s.c.) tumors were grown in both immune-competent (C57BL/6) (Figure 4) and incompetent (nude) (Figure 5) animals. The effect of LDH-A KD on corresponding growth profiles of s.c. flank tumors in immune-competent C57BL/6 mice are shown (Figure 4E–G), and the tumor doubling times were estimated (Figure 4H). As shown in the in vitro studies, LDH-A KD had a significant effect only on the growth of s.c. GL261 tumors in C57BL/6 mice (Figure 4E). All s.c. GL261 LDH-A KD tumors regressed after developing small (<50 mm^3^) tumors, whereas most GL261 NC tumors grew after a long 40-day delay period (Figure 4E). Once GL261 NC tumors began to grow in C57BL/6 mice, the subsequent doubling time was similar to that of wild-type GL261 tumor growth in C57BL/6 mice (data are not shown). No significant difference was observed between the growth profiles of the NC and KD groups of CT2A and ALTS1C1 tumors in C57BL/6 mice (Figure 4F–H). Wild-type CT2A and ALTS1C1 tumors grew slightly faster than NC tumors in C57BL/6 mice, but the difference was not statistically significant (data not shown). These results suggest that the LDH-A knock-down did not significantly alter the growth of s.c. CT2A and ALTS1C1 tumors growth in C57BL/6 mice compared with NC control tumors, but had a clear effect on GL261 tumor growth in C57BL/6 mice—leading to tumor regression. This observation is similar to the growth delay and inhibition we observed in 4T1 orthotopic murine breast tumors in immune-competent and immune-suppressed host animals, following LDH-A KD [12].

In immune-compromised nude mice, the growth and doubling times of NC and LDH-A KD GL261, CT2A and ALTS1C1 tumors were similar (Figure 5A–D). Interestingly, GL261 NC tumors grew at a faster rate (doubling time, 3.1 ± 1.5 days) in nude mice than GL261 NC tumors in C57BL/6 mice (7.1 ± 0.3 days) (*p* = 0.002) (Figure 5E). The difference between host animals was reversed with ALTS1C1 NC and CT2A LDH-A KD tumors; tumors grew more slowly in nude compared to C57BL/6 mice. These data demonstrate the variability of NC and LDH-A KD tumor growth in host animals with different tumor microenvironments and immune responses.

### 3.5. LDH Isoenzyme Pattern of Subcutaneously Located Murine Glioblastoma Tumors

**Figure 6:**
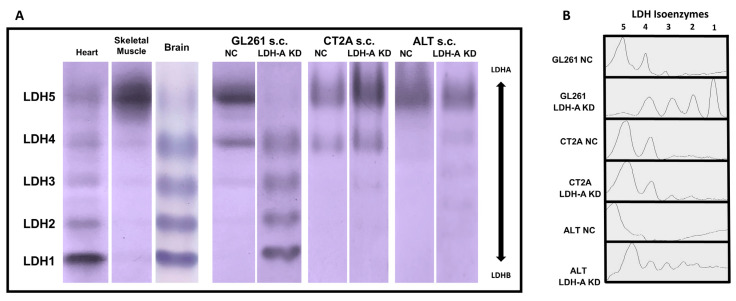
**Native-polyacrylamide gel electrophoresis LDH zymograms for ex vivo tissue and s.c. GL261, CT2A and ALTS1C2 tumors.** Electrophoretic patterns in the heart and skeletal muscle as well as s.c. tumors from NC and LDH-A KD GL261, CT2A and ALTS1C1 tumors (Panel **A**), corresponding LDH isoform profiles (Panel **B**); *n* = five independent studies.

LDH zymography is a common method to detect LDH isoenzymes and provides for the direct observation of five isozyme bands in the active state [34]. The LDH isoenzyme pattern for GL261, CT2A and ALTS1C1 LDH-A KD and NC subcutaneous tumors were compared with each other and to heart and skeletal muscle tissue from the same animals (Figure 6A,B). 

All NC tumors have an isoenzyme pattern similar to skeletal muscle (LDH5- and LDH-A-dominant), with some formation of LDH 4, 3 and 2. CT2A and ALTS1C1 LDH-A KD tumors have a similar LDH isoenzyme pattern as the NC tumors. Only GL261 LDH-A KD tumors were strikingly different, with a LDH isoenzyme pattern similar to the heart, where LDH1, 2, 3 and 4 isoenzymes are most highly expressed [43]. The LDH isoenzyme ratio of the brain tissue was comparable to ratios found in the heart tissue that present mostly the isoenzymes LDH1-LDH3 and low amounts of the LDH5 isoform [43]. These results are consistent with the mRNA and Western blot assays and calculated LDH-B/LDH-A ratios (Figure 1). 

### 3.6. LDH-A and LDH-B Expression Patterns in Tumors

**Figure 7:**
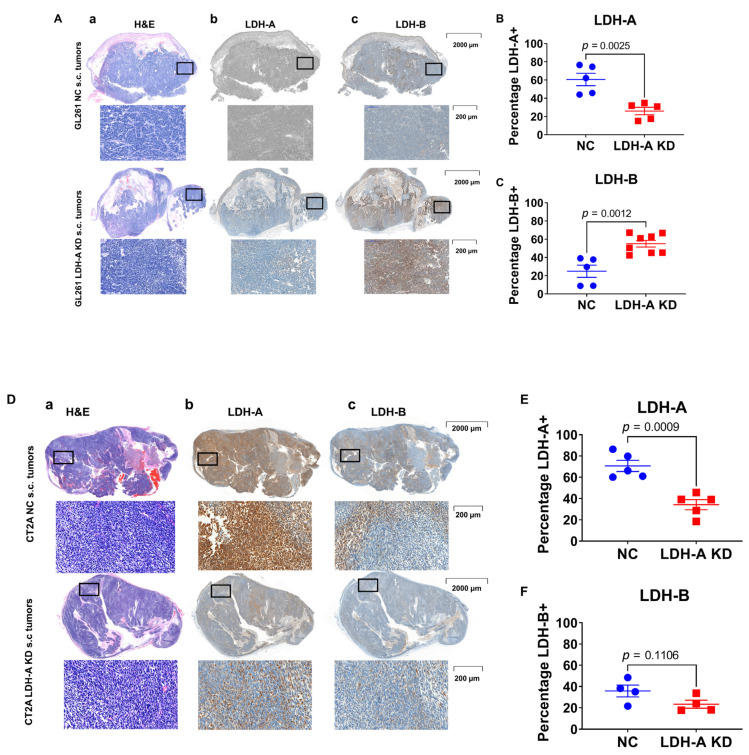
**H&E and IHC staining for LDH-A and LDH-B protein expression in s.c. GL261 and CT2A tumors—both LDH-A KD and NC controls**. H&E staining for GL261 NC and LDH-A KD tumors (**Aa**); LDH-A staining (**Ab**) and LDH-B staining (**Ac**). GL261 NC and LDH-A KD tumors were grown in immunocompromised nude mice. Quantification of percentage LDH-A tumor expression (**B**) and percentage LDH-B tumor expression (**C**); ±SEM. A similar presentation is shown for CT2A tumors, grown in immune-competent C57BL/6 mice (Panels **D**–**F**).

First, we assessed the structure (by H&E staining) of subcutaneous GL261 NC and KD tumors growing in nude mice (GL261 KD tumors did not grow in immune-competent C57BL/6 mice) and that of CT2A NC and KD tumors growing in C57BL/6 mice (Figure 7). We chose to focus on GL261 and CT2A tumors because of their similar mRNA, Western blot, and Seahorse metabolic profiles, in comparison to that of ALTS1C1 (Figure 1, Figure 2 and Figure 3A). Moreover, RNAseq data confirmed the absence of any difference in *LDH-A* and *LDH-B* levels between control and knock-down ALTS1C1 cells (Figure 3A). There was a variable pattern of necrosis, stroma and cyst formation for both tumors (Figure 7(Aa,Da)). Second, LDH-A and LDH-B immunohistochemistry also showed a variable pattern of staining for both types of tumors (Figure 7(Abc,Dbc)). A Weka analysis [31] showed significantly greater LDH-A staining in both GL261 and CT2A NC tumors compared to the LDH-A KD tumors (Figure 7B,E, Table S1), consistent with Western blotting and LDH enzymatic assay results (Figure 1C,E,F). Considerably greater LDH-B staining was observed in GL261 LDH-A KD compared to NC tumors (Figure 7C, Table S1), but no differences in LDH-B staining were observed between CT2A LDH-A KD and NC tumors (Figure 7F, Table S1), also consistent with Western blotting results (Figure 1D,E,H).

In many, but not all, GL261 tumor regions, there was an inverse relationship between LDH-A and LDH-B expression intensity (Figures S2A and S3A). This inverse relationship was greater for LDH-A KD than NC GL261 tumors (Figure S3A). CT2A tumors showed a different relationship; there was a more direct relationship between LDH-A and LDH-B expression intensity (Figures S2B and S3B).

## 4. Discussion

It has been shown that that LDH-A-associated lactic acid accumulation in melanoma inhibits tumor surveillance by T and NK cells, and that elevated levels of lactic acid prevent the upregulation of the nuclear factor of activated T cells (NFAT) in T and NK cells, resulting in diminished IFN-γ production [44]. These results demonstrate that lactic acid can be a potent inhibitor of T and NK cell function and survival, leading to tumor-immune escape. We previously reported that the downregulation of LDH-A expression in 4T1 murine breast cancer cells in vitro and in 4T1 tumors located in the mammary fat pad leads to reduced glycolytic flux and increased mitochondrial respiration, leading to a slower growth and the delayed onset of (or failure to develop) distant metastases in both immune-compromised mice [11] and in immune-competent mice [12]. Here, we compare three murine glioma cell lines (GL261 [27], CT2A [30] and ALTS1C1 [29]) and their growth in both immune-competent (C57BL/6) and immune-compromised (athymic Foxn/nu or *nu*/*nu*) mice. Our objective was to better understand the impact of LDH-A downregulation (KD) on glioma tumor phenotype and growth potential in a subcutaneous location. In an accompanying manuscript [26], we compare the same cell lines and the intracranial growth of these tumors, focusing on a comparison of LDH-A depletion by shRNA knock-down and by the pharmacologic inhibition of LDH.

Here, we found that the growth of s.c. GL261 LDH-A KD tumors regressed after developing small (<50 mm^3^) tumors, whereas most GL261 NC tumors grew after a long 40-day delay period. No significant difference was observed between the s.c. growth profiles of the NC and KD groups of CT2A and ALTS1C1 tumors in C57BL/6 mice. The differences and similarities in the growth profiles between the six cell lines are consistent with the differences and similarities observed in their mRNA, LDH zymogram, enzymatic and Western blot profiles. Our observations in GL261 LDH-A KD and control NC tumors are more similar to the growth delay and inhibition we observed in 4T1 orthotopic murine breast tumors in immune-competent and immune-suppressed host animals, following LDH-A KD [12]. However, the difference in growth profiles of GL261 tumors compared to ALTS1C1 or CT2A s.c. tumors in C57BL/6 mice cannot be explained by a blunting of immunosurveillance due to high LDH-A expression, since LDH-A mRNA, protein and enzyme activity were the lowest in GL261 cells (compared to that in ALTS1C1 or CT2A cells tumors).

The major distinguishing characteristics of the GL261 LDH-A knock-down cells and tumors (compared to ALTS1C1 or CT2A) include a high level of LDH-B and the ability of GL261 LDH-A KD cells (and most likely tumors) to accumulate intracellular lactate, due in part to the downregulation of the MCT 4 transporter and the ability to convert lactate to pyruvate and further processing due to the up-regulation of the oxidative phosphorylation pathway. Only the GL261 LDH-A KD cell line showed a higher expression of LDH-B mRNA, and higher LDH-B/LDH-A mRNA and protein ratios compared to the other five cell lines. Additionally, control GL261 NC cells had the lowest levels of LDH-A and LDH-B, and LDH enzyme activity of the three control cell lines. The metabolic profile of these cells, using Seahorse XF technology, showed a limited energetic phenotype of GL261 glioma (with the lowest ATP production) compared to CT2A and ALTS1C1 cells, thereby linking the LDH activity in GL261 cells with its observed metabolic phenotype.

LDH is the enzyme catalyzing the final step of glycolysis and contains two subunits, A and B, encoded by two genes [45]. LDH-A is predominantly found in skeletal muscle and LDH-B is predominantly expressed in the heart and brain. LDH-A and LDH-B can form homo- or hetero-tetramers, forming five LDH isoenzymes: LDH-1 (4B), LDH-2 (3B,1A), LDH-3 (2A, 2B) and LDH-5 (4A) [46]. These five isoforms catalyze the same overall reaction but differ in their affinity to the substrate, inhibition concentration (Km), isoelectric point and electrophoretic mobility. The five isoforms can be visualized in the active state using LDH zymography [47]. A zymogram analysis of the six glioma cell lines was performed and showed an LDH-A-dominant pattern for five of the six cell lines (containing mostly LDH5 and LDH4 isoenzymes). Only GL261 LDH-A KD cells showed an LDH-B-dominant pattern (containing mostly LDH1, LDH2 and some LDH3, LDH4 isoenzymes). This major shift in the LDH isoenzyme pattern in GL261 LDH-A KD tumors, (from LDH-A-dominant in NC tumors to LDH-B-dominant in LDH-A KD tumors), can lead to differences in the kinetics of the LDH oxidative vs. reductive enzyme activity and in cell metabolism [47,48]. Similar variations in LDH-A and LDH-B isoforms have been detected in human glioma cells D54MG and U-251MG [6], but this difference was not explored in detail, or related to tumor growth, metabolism and phenotype.

The LDH-A and LDH-B immunohistochemistry and Weka analysis confirmed the isoenzyme patterns observed with LDH zymography. GL261 and CT2A KD tumors showed significantly less LDH-A staining than their control NC tumors. Additionally, the significantly greater LDH-B staining, only in GL261 LDH-A KD, was consistent (compared to NC tumors); no significant differences in LDH-B staining was observed between CT2A LDH-A KD and NC tumors. We also noted an “inverse” LDH-A/LDH-B staining relationship (high vs. low) in many, but not in all GL261 tumor regions. In contrast, CT2A tumors showed a more “direct” LDH-A/LDH-B staining relationship (high vs. low) in many tumor regions. These results are consistent with the known spatial heterogeneity of gliomas [49,50]. The differences in LDH isoenzyme patterns and LDH-A/LDH-B immunohistochemistry were also reflected in subcutaneous tumor growth experiments. First, LDH-A KD prolonged the doubling time of GL261 cells in nutrient rich culture media. Second, GL261 LDH-A KD cells did not establish flank tumors in immune-competent C57BL/6 mice, whereas GL261 NC tumors formed after a 40-day growth delay, indicating that the metabolic rearrangements of GL261 LDH-A KD cells did not support s.c. tumor growth in immune-competent C57BL/6 mice. Third, both NC and KD GL261 tumors grew more rapidly in nude mice, compared to GL261 NC tumors growing in C57BL/6 mice. These results show the combined impact of both a metabolic alteration (LDH-A KD) and the immune system (C57BL/6 vs. nude mice) on the growth of s.c. located tumors. Furthermore, the ability to grow GL261 tumors in nude mice allowed us to compare the isoenzyme profiles of LDH-A KD and NC tumors using zymogram analyses.

The association of LDH-A and lactate with cancer metabolism and tumor growth has been studied extensively, including that in human gliomas [7,47,48,49]. Furthermore, the role of LDH-A has been shown to be quite different in different tumors [9,51,52,53,54]. For example, the studies by Chesnelong et al. established that LDH-A was under-expressed in different grades of IDH mutated gliomas, where low expression of LDH-A and high methylation of the LDH-A promoter was found in IDHmt glioblastoma (GBM) patients [23]. Glioma patients with tumors with over-expressed LDH-A had a median survival of 16 months, whereas patients with tumors with under-expressed LDH-A had a median survival of >50 months [23]. These authors suggested that the silencing of LDH-A in gliomas with IDH mutations may be responsible in part for their characteristically slow progression. Although LDH-A is highly expressed in many tumors, the role of LDH-A and lactate has also been shown to vary in different tumors [9,51,52,53,54]. The association of LDH-B with tumors is much more complex [15]. Although a high expression pf LDH-B is not present in many tumor types [15], it has been shown to play a significant role in the metabolism of some tumors [55,56,57].

It has been suggested that glioma cells express one of at least two different metabolic phenotypes [6,58], as reflected in their different metabolic profiles. The 251MG and U-87MG glioma cells have been shown to have metabolic characteristics similar to astrocytes (that include the production of lactate, the storage of glycogen and the use of lactate to support neurons). These tumors exhibit a glycolytic-dependent phenotype, but retain functional oxidative phosphorylation and primarily express LDH-B. In contrast, GL261 and D-54MG glioma and SH-SY5Y neuroblastoma cells display a more oxidative phosphorylation-dependent phenotype, and express both LDH-A and LDH-B isoforms. Therefore, we hypothesized that LDH-A knock-down could cause a shift in the metabolic phenotype of GL261 cells (but not CT2A and ALTS1C1 cells, which remain LDH-A-dominant).

Our RNA-seq bioinformatics analysis provides additional evidence that GL261 LDH-A KD cells may have an improved ability to convert lactate into pyruvate and entry into the TCA cycle. A Gene Set Enrichment Analysis (GSEA) showed that GL261 KD cells had a significant enrichment of the oxidative phosphorylation pathway, but little or no change in the expression of glycolytic genes (with the exception of PGK1). Neither CT2A or ALTS1C1 LDH-A-depleted cells showed any consistent enrichment of either glycolytic or oxidative pathway genes when compared to their respective NC controls. In addition to decreased LDH-A and increased LDH-B, GL261 KD cells also showed a significant downregulation of the lactate exporter MCT4 (*SCL16A3*). We also observed that GL261 cells (both NC and KD, compared to the other cell lines) had the highest levels of PDHA1 (pyruvate dehydrogenase E1 component subunit alpha), an enzyme which catalyzes the conversion of pyruvate into acetyl-CoA in the mitochondria, and feeds acetyl-CoA into the TCA cycle. This suggests that GL261 glioma cells are pre-programed to have the capacity for high TCA cycle activity and that this capacity is enhanced by LDH-A depletion with the predominance of LDH-B expression. The data suggest that GL261 KD cells also have the ability to reprogram their transcriptome to support alternative (non-glycolytic) metabolic pathways in LDH-A-depleted cells. Furthermore, it suggests that GL261 LDH-A-depleted cells, through their unique upregulation of LDH-B, may be able to metabolize lactate into pyruvate and through the TCA cycle.

The high maximum respiration capacity detected in GL261 LDH-A KD cells (following the addition of the uncoupler FCCP), demonstrates the magnitude of physiological “energy demand” which can be achieved by rapid oxidation of substrates (sugars, fats and amino acids) to meet this metabolic challenge. We and others [11,48] suggested that ATP production in cells by upregulated OXPHOS may be associated with greater proton leak and ROS (reactive oxygen species) production and may lead to cell death. This is consistent with prior findings showing that the inhibition of LDH-A induces oxidative stress and inhibits tumor progression [48]. The major distinguishing characteristic of the GL261 cells and tumors (compared to ALTS1C1 or CT2A) were a high level of LDH-B and the ability of GL261 cells to upregulate the oxidative phosphorylation pathway. These differences are accentuated following LDH-A KD in immunocompetent mice (of note—this change was present, but less prominent in nude mice). ROS produced by cellular metabolism can also play an important role as signaling messengers in immune system. It is known that elevated ROS in the tumor microenvironment is associated with tumor-induced immunosuppression [59]. These results illustrate that there are both metabolic and phenotypic differences between glioma cell lines and tumors that impact tumor growth.

## 5. Conclusions

Six genetically altered murine glioma cell lines and tumors (with LDH-A shRNA knock-down (KD) vs. scrambled shRNA controls—NC) were compared for differences in their levels of LDH-A and LDH-B mRNA, protein and enzymatic activity, as well as their glycolytic and oxidative metabolic pathway activity. An RNA sequencing (RNASeq) analysis and the above studies showed that GL261 glioma cells (but not CT2A and ALTS1C1 cells) are pre-programmed to have the capacity for high TCA cycle activity and that this capacity is enhanced by LDH-A depletion. The expression of glycolytic pathway genes was not significantly affected by LDH-A KD. These results show the combined impact of LDH-A depletion (LDH-A KD vs. NC control) and the immune system (C57BL/6 vs. nude mice) on the growth of s.c. located tumors and suggest that GL261 glioma cells (but not CT2A and ALTS1C1 cells) can adopt different metabolic pathways with higher TCA cycle activity under environmental stress, and that this capacity is enhanced by LDH-A depletion.

## Data Availability

Table S2 of the Supplement provides the semi-raw data from the RNA Seq analysis that was used to generate the figures. The Blasberg Lab has closed at MSKCC, although relevant data exists on the MSK servers that can be accessed by Dr. Blasberg, he currently is an Emeritus Professor.

## Supplementary Materials

The following supporting information can be downloaded at: https://www.mdpi.com/article/10.3390/cancers14092303/s1, Figure S1: Characterization of control (NC) murine glioma cell lines (GL261, CT2A, ALTS1C1) upon LDH-A shRNA knockdown; Figure S2: Local IHC staining for LDH-A and LDH-B proteins in s.c. GL261 and CT2A, NC and LDH-A KD tumors; Figure S3: Local intratumoral LDHA vs LDH-B protein expression relationships in s.c. GL261 and CT2A, NC and LDH-A KD tumors; Figure S4: Native western blots of Panel E in Figure 1; Table S1: Weka analysis for ICH Staining for LDH-A and LDH-B of s.c. GL261 and CT2A tumors; Table S2: Semi-raw data from the RNASeq analysis that was used to generate Figure 3.
