# Supplementary material for: LDH-A—Modulation and the Variability of LDH Isoenzyme Profiles in Murine Gliomas: A Link with Metabolic and Growth Responses
Source: Cancers (Basel). 2022 May 6;14(9):2303. doi: 10.3390/cancers14092303 (PMC9100845; doi:10.3390/cancers14092303)
Supplement: Supplementary file 1 [file cancers-14-02303-s001.zip › cancers-1518197-supplementary/Supplementary Information (File1) - cancers-1518197.pdf]

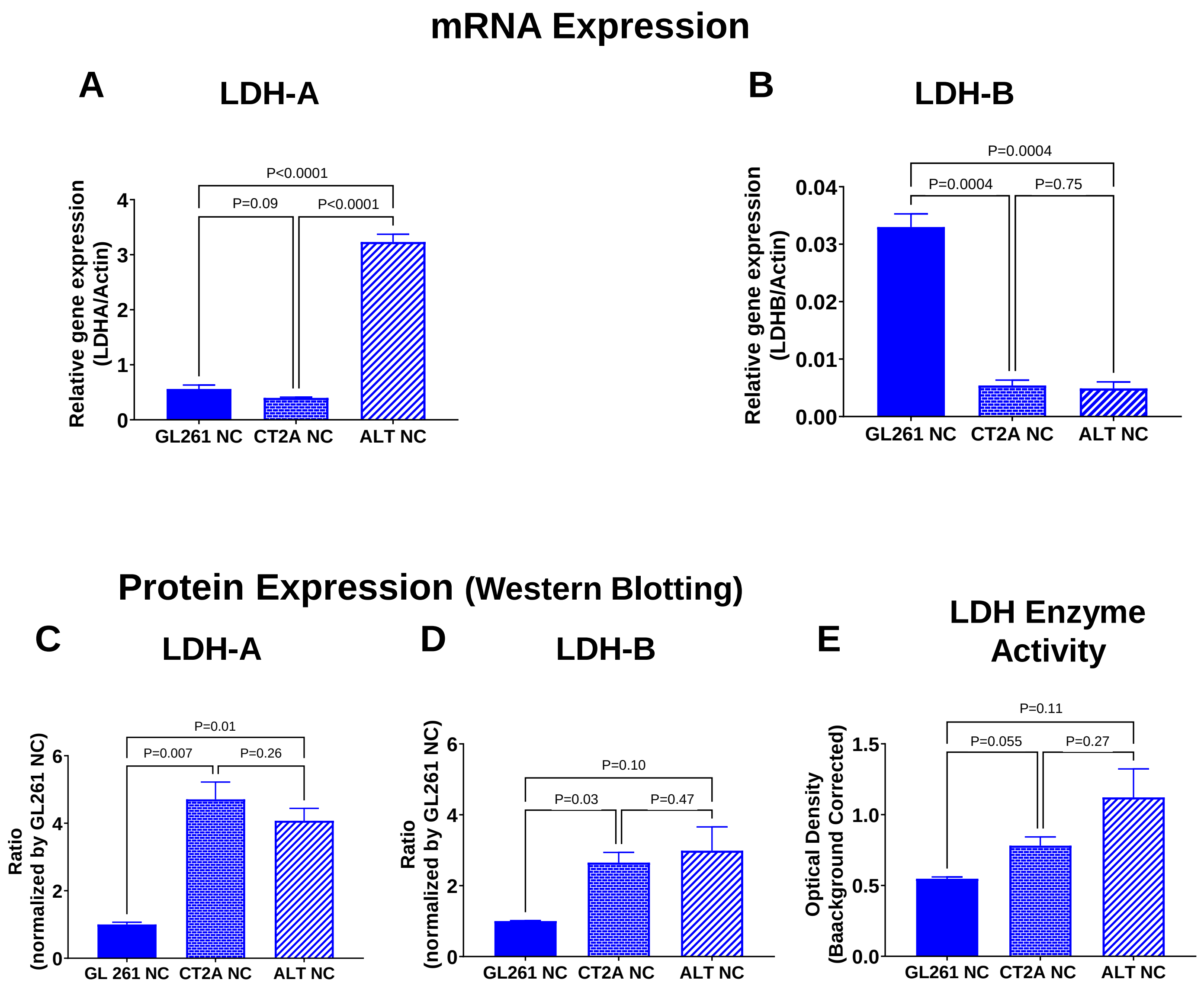

Figure S-1. Characterization of control (NC) murine glioma cell lines (GL261, CT2A, ALTS1C1) upon LDH-A shRNA knockdown (n=3 for all data presented).

Table S-1: Weka analysis for IHC Staining for LDH-A and LDH-B of s.c. GL261 and CT2A tumors.

	GL261 (nude mice)		CT2A (CB57BL/6 mice)	
	NC	LDH-A KD	NC	LDH-A KD
LDH-A+ % of tumor	60.6 ± 15.4	26.1 ± 9.1	70.7 ± 11.8	32.2 ± 10.7
P value	0.002		0.0009	
LDH-B+ % of tumor	24.9 ± 14.9	55.2 ± 10.4	35.9 ± 11.1	23.4 ± 7.5
P value	0.001		0.11	

Table summarizes the Weka analysis shown in Figure 4 (Panels B, C, E, F).

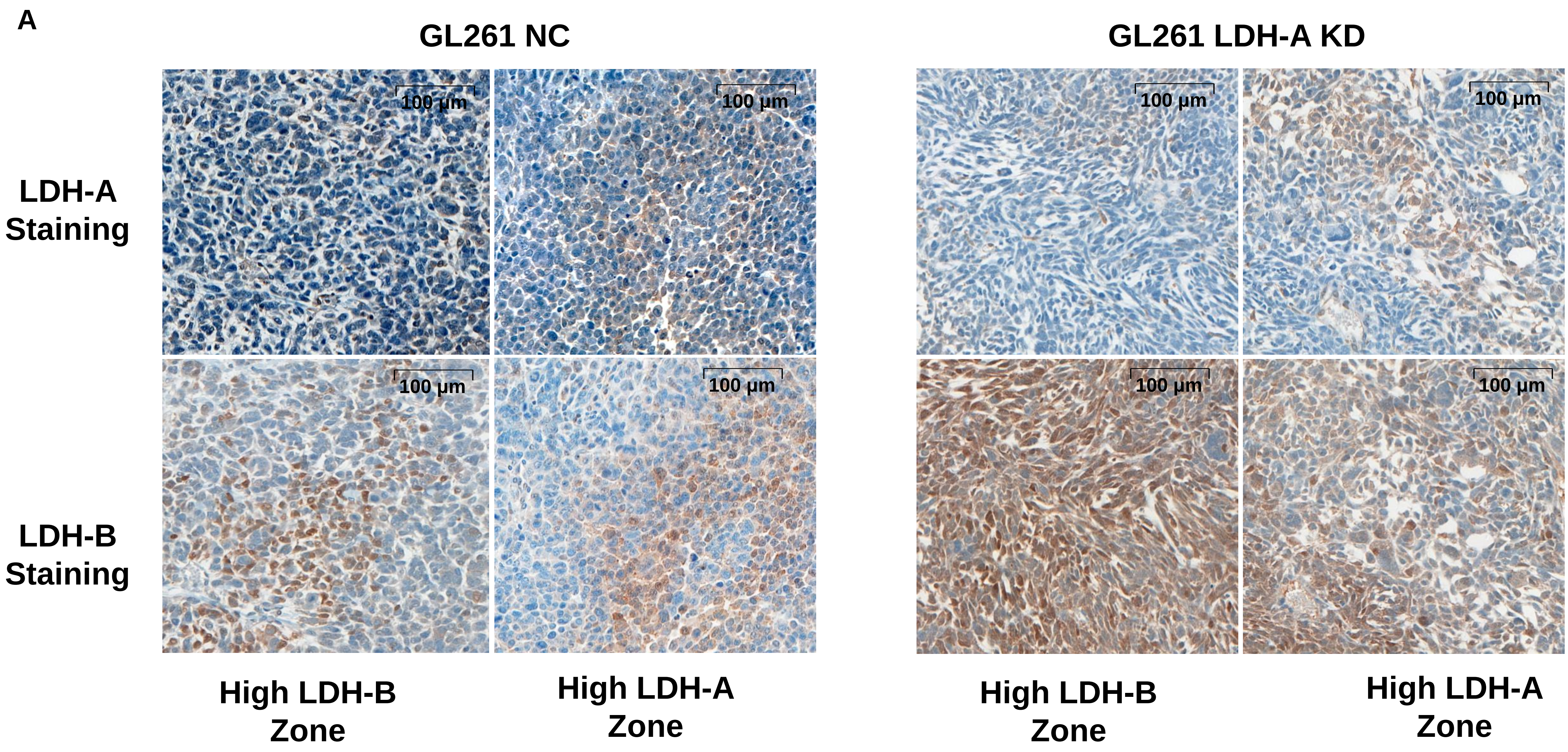

Figure S-2A. Local IHC staining for LDH-A and LDH-B proteins in s.c. GL261 NC and LDH-A KD tumors.

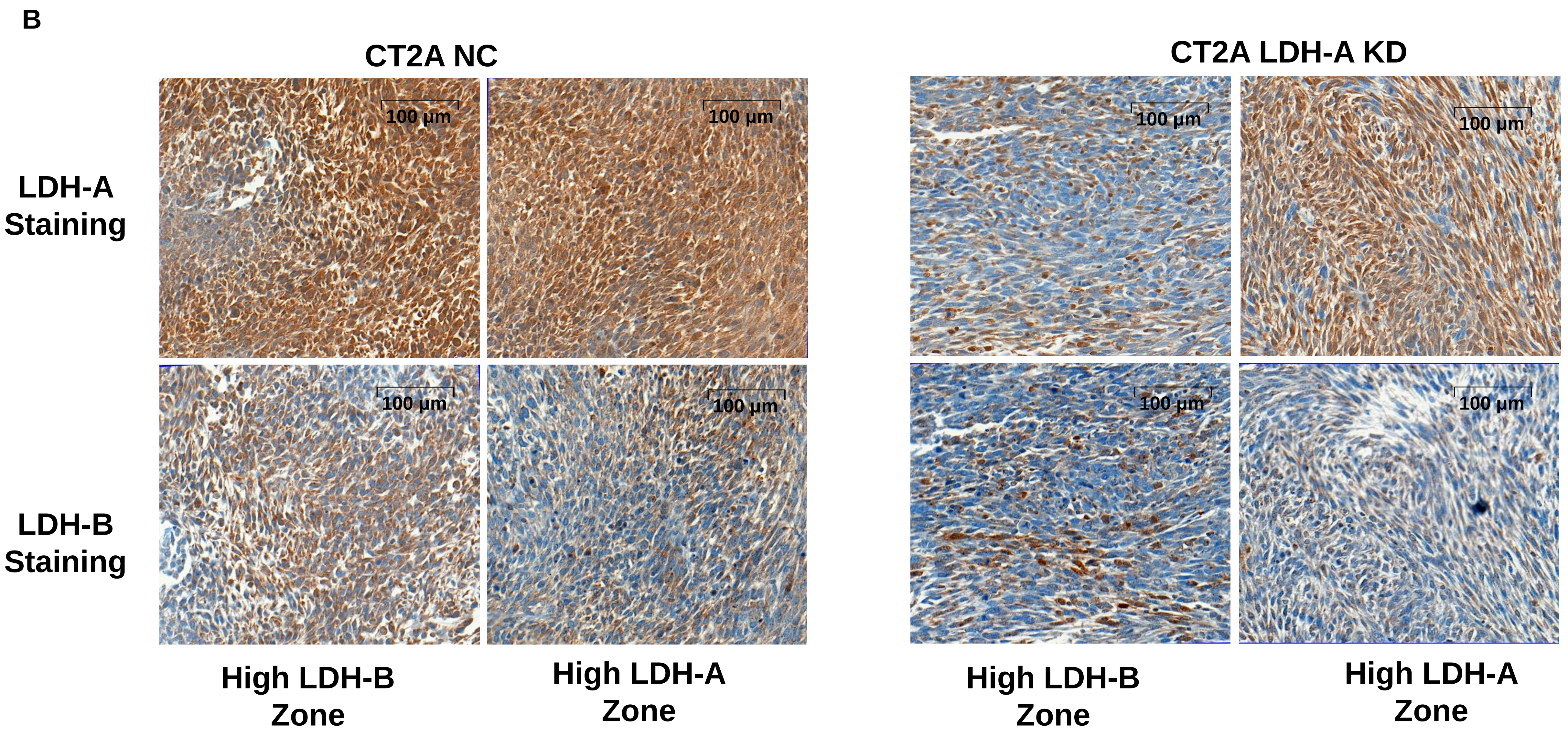

Figure S-2B. Local IHC staining for LDH-A and LDH-B proteins in s.c. CT2A tumors.

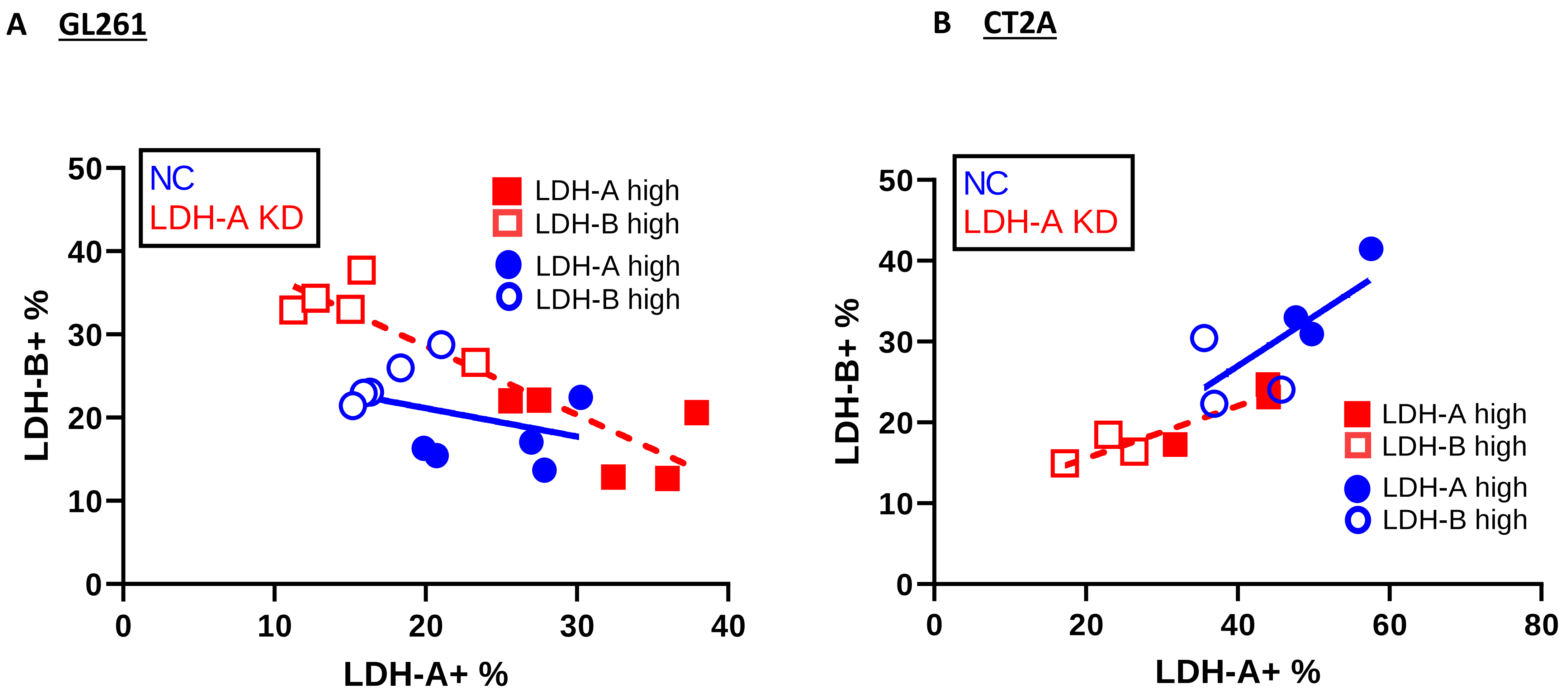

Figure S-3. Local intratumoral LDH-A vs LDH-B protein expression relationships in s.c. GL261 and CT2A, NC and LDH-A KD tumors. An inverse local staining intensity relationship between LDH-A and LDH-B was observed in many GL261 tumor regions **(A)**, whereas a more direct relationship between LDH-A and LDH-B staining intensity was observed in many local CT2A local tumor regions **(B)**.

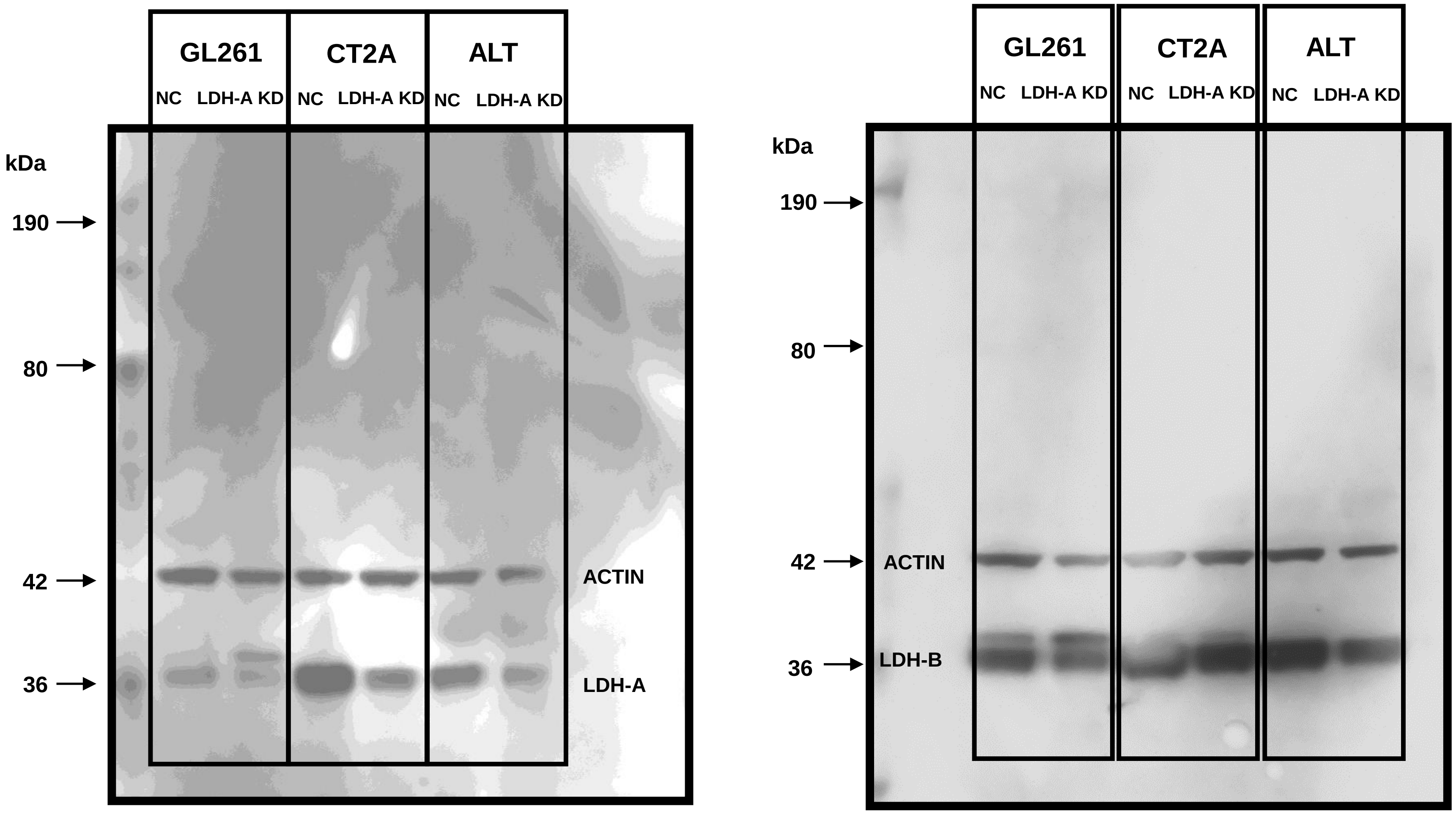

Figure S-4. Native western blots of Panel E in Figure 1.
